# Long-term cognitive follow-up in children treated for Maroteaux-Lamy syndrome

**DOI:** 10.1007/s10545-015-9895-8

**Published:** 2015-10-08

**Authors:** Berendine J. Ebbink, Marion M. G. Brands, Johanna M. P. van den Hout, Maarten H. Lequin, Robert R. J. Coebergh van den Braak, Rianne L. van de Weitgraven, Iris Plug, Femke K. Aarsen, Ans T. van der Ploeg

**Affiliations:** Center for Lysosomal and Metabolic Diseases, Department of Pediatrics, Erasmus MC University Medical Center — Sophia Children’s Hospital, P.O. Box 2060, 3000 CB Rotterdam, The Netherlands; Department of Pediatric Neurology, Erasmus University Medical Center — Sophia Children’s Hospital, Rotterdam, The Netherlands; Department of Radiology, Erasmus University Medical Center, Rotterdam, The Netherlands; Department of Pediatrics, Erasmus University Medical Center — Sophia Children’s Hospital, Rotterdam, The Netherlands

## Abstract

**Background:**

It remains unclear to what extent the brain is affected by Maroteaux-Lamy syndrome (MPS VI), a progressive lysosomal storage disorder. While enzyme replacement therapy (ERT) elicits positive effects, the drug cannot cross the blood–brain barrier. We therefore studied cognitive development and brain abnormalities in the Dutch MPS VI patient population treated with ERT.

**Methods:**

In a series of 11 children with MPS VI (age 2 to 20 years), we assessed cognitive functioning and brain magnetic resonance imaging prospectively at the start of ERT and at regular times thereafter up to 4.8 years. We also assessed the children’s clinical characteristics, their siblings’ cognitive development, and their parents’ educational levels.

**Results:**

The patients’ intelligence scores ranged from normal to mentally delayed (range test scores 52–131). In 90 %, their scores remained fairly stable during follow-up, generally lying in the same range as their siblings’ test scores (median for patients = 104, median for siblings = 88) and comparing well with the parental educational levels. Native-speaking patients had higher intelligence test scores than non-native-speaking patients. Two patients, both with high baseline glycosaminoglycan levels in their urine and severe mutations in the arylsulfatase B gene, scored clearly lower than expected. Patients with pY210C performed best. Brain abnormalities were aspecific, occurring more in patients with severe symptoms.

**Conclusion:**

Our study shows that cognitive development in MPS VI patients is determined not only by familial and social-background factors, but, in patients with a severe form of the disease, also by the disease itself. Therefore in patients with severe disease presentation cognition should be monitored carefully.

**Electronic supplementary material:**

The online version of this article (doi:10.1007/s10545-015-9895-8) contains supplementary material, which is available to authorized users.

## Introduction

Maroteaux-Lamy syndrome (MPS VI, OMIM #253200) is a lysosomal storage disease caused by a deficiency of the enzyme arylsulfatase B, which leads to storage of dermatan sulphate (a glycosaminoglycan, GAG). Its characteristic clinical features include short stature, bone abnormalities, sensory perception disorders, corneal clouding, carpal tunnel syndrome, spinal cord compression, and reduced life expectancy (Azevedo et al. 2004; Giugliani et al. 2007; Neufeld and Muenzer 2001; Spranger et al. 1970; Stumpf et al. 1973; Valayannopoulos et al. 2010). The disease spectrum varies from a rapidly progressive form, to milder variants with later onset of symptoms. Disease severity is related to urinary GAG levels (Brands et al. 2013a, b; Giugliani et al. 2007; Swiedler et al. 2005; Valayannopoulos et al. 2010). In 2007, enzyme replacement therapy (ERT) with recombinant human arylsulfatase B was registered as a treatment for MPS VI. Although this has positive effects on various tissues (Brands et al. 2013a, b; Decker et al. 2010; Harmatz et al. 2008; Harmatz et al. 2010), it is assumed that ERT cannot pass the blood–brain barrier. While progressive mental retardation is common in other types of mucopolysaccharidoses (Neufeld and Muenzer 2001), reports on cognition in MPS VI patients are inconsistent (Azevedo et al. 2013; Spranger et al. 1970; Vestermark et al. 1987). Several reports have indicated that cognition in MPS VI patients is normal. A recent cross-sectional study among Brazilian patients reported mental retardation in one third of the patients, but also indicated that severe visual and/or hearing deficits might have influenced the test results (Azevedo et al. 2013). Many patients had brain abnormalities.

We prospectively studied the mental development and MRI findings in the Dutch MPS VI patient population to increase insight into 1) long-term cognitive outcome, 2) the potential influence of social and familial background on intelligence, 3) cognitive outcome relative to the urinary GAG levels and gene mutations, and 4) the potential relationship between structural abnormalities of the brain and cognition.

## Methods

### Patients

All Dutch patients with Maroteaux-Lamy syndrome treated with ERT participated in this long-term standardized follow-up study, which was performed at the Center of Lysosomal and Metabolic Diseases at Erasmus MC University Medical Center in Rotterdam. MPS VI was diagnosed on the basis of GAG analysis in the urine followed by enzyme assay on leukocytes and fibroblasts, and then by mutation analyses in the Arylsulfatase B gene (Brands et al. 2013a, b). Patients were treated with the registered dose of 1 mg/kg/weekly recombinant human arylsulfatase B (galsulfase, Naglazyme®, Biomarin Corporation). None had received hematopoietic stem-cell transplantation.

Study protocols were approved by the Institutional Review Board. Written informed consent was provided by all patients and siblings participating in this study, if necessary in conjunction with their parents.

### Intelligence

Each year, patients underwent standardized cognitive assessments, for which we used the following: the Griffith Mental Development Scales (Griffiths 1984, 1986); the Bayley Scales of Infant Development (van der Meulen et al. 2002); and the Wechsler Intelligence Scales (Wechsler Intelligence Scales Children-third edition; 6–16 years, or Wechsler Adult Intelligence Scales-third edition; > 16 years) (Kort et al. 2005). For children with impaired hearing, we used the Snijders-Oomen Nonverbal Intelligence Test-Revised (SON-R 2½-7) (Tellegen et al. 2008).

To investigate social and familial background, we compared each patient’s intelligence test score with that of their sibling or siblings. Patients and siblings were tested by three pediatric neuropsychologists (F.K.A., B.J.E., and R.L.v.d.W.). We also collected information on the parents’ native language and highest educational level, and on patients’ and siblings’ school performance.

### Brain MRI

Brain magnetic resonance imaging (MRI) was performed regularly, and the baseline MRI and most recent MRI were used to determine change over time. The choice of parameters was based on literature review, being deduced particularly from four articles on brain abnormalities in patients with MPS VI (see Supplement) (Azevedo et al. 2013; Gabrielli et al. 2004; Lee et al. 1993; Seto et al. 2001). Two observers evaluated all MRIs on T2, FLAIR sequence: a physician assistant trained for this job (M.B.), and a pediatric neuroradiologist (M.H. L.).

### Statistics

Performances in the psychological tests were compared against the normative data of the Dutch population. The mean score for each of these tests is 100, with a standard deviation (SD) of 15 points. A score above 84 reflects normal development, a score between 84–70 indicates mild developmental delay, and a score below 70 severe developmental delay. Per patient, a difference was defined as a deviation of more than 1.5 standard deviations between total intelligence test scores (Lezak et al. 2012). The Mann–Whitney *U* test to determine differences between groups was used. All analyses were performed with SPSS for Windows (version 20, SPSS Inc., Chicago, IL).

## Results

### Patients characteristics

Eleven children with MPS VI were included in this study; among them were two sibling couples. Patients were of Dutch (4), Turkish (4), Moroccan (1), Pakistani (1) and Guinean (1) ancestry. Seven had consanguineous parents.

Patient characteristics are summarized in Table 1.

**Table 1:** Patient characteristics

Patient	Gender	Age at first symptoms (years)	Age at diagnosis (years)	Age at start of treatment (years)	Allele 1	Allele 2	Rapid/slow progressive	GAG-level at baseline (upper limit of normal for age)	Hearing aids ^2^	Glasses ^3^	Native speaker	Mother’s education	Father’s education	Consanguine	Carpal tunnel release
1^1^	M	5.0	0.7	7.6	454 C > T (p.R152W), exon 2	454 C > T (p.R152W), exon 2	Slow	231 (318)	N	Y	N	1	1	Y	N
2	F	1.5	1.8	2.1	903 C > G (p.N301K) exon 5, S384N, 1152 G > A, exon 6	903 C > G (p.N301K) exon 5, S384N, 1152 G > A, exon 6	Rapid	942 (254)	Y	Y	N	4	1	N	Y
3	M	.6	1.9	2.3	971 G > T (p.G324V), exon 5	971 G > T (p.G324V), exon 5	Rapid	739 (254)	N	Y	N	1	2	N	Y
4	M	1.2	2.8	3.0	995 T > G (p.V323G), exon5	995 T > G (p.V323G), exon5	Rapid	554 (194)	Y	Y	N	4	4	Y	N
5	F	.4	3.4	6.7	c.1142 + 2 T > C, exon 5	c.1142 + 2 T > C, exon 5	Rapid	1287 (194)	Y	Y	N	1	2	Y	Y
6	M	.8	5.1	5.9	629 A > G (p.Y210C), exon 3	979C > T (p.R327X)	Slow	207 (145)	Y	N	Y	4	4	N	Y
7^1^	M	3.0	5.8	6.2	629 A > G (p.Y210C), exon 3	979C > T (p.R327X)	Slow	158 (145)	N	Y	Y	6	5	Y	N
8	F	3.0	7.4	7.8	629A > G (p.Y210C), exon 3	979C > T (p.R327X)	Slow	214 (124)	N	N	Y	6	5	Y	N
9	F	2.0	7.8	8.3	454C > T (p.R152W), exon 2	454C > T (p.R152W), exon 2	Slow	254 (124)	N	Y	N	3	2	Y	N
10	M	7.0	10.1	18.3	454 C > T (p.R152W), exon2	454 C > T (p.R152W), exon2	Slow	106 (107)	N	N	N	1	1	Y	Y
11	M	9.0	10.2	10.7	629 A > G (p.Y210C)	937 (C > G) (p.P313A)	Slow	193 (107)	N	N	Y	6	5	N	N

M = male, F = Female, N = No, Y = Yes, 1 = Elementary School, 2 and 3 = Junior High School, 4 = Senior High School, 5 = Bachelor of Science, 6 = Master of Science

^1^ diagnosed after the diagnosis of sibling (patient 1 is the sibling of patient 10; patient 7 is the sibling of patient 8)

^2^ conductive hearing loss (range 7–43 dB right ear; 2–53 dB left ear) (Brands et al. 2013b)

^3^ hyperopia

The median age at presentation of symptoms was 2.0 years; at diagnosis it was 5.1 years, and at baseline (start of enzyme therapy) it was 6.8 years. One patient was assessed at baseline only (patient 11). The other patients had at least a follow-up of two years (three assessments) or longer, range 0–4.8 years.

### Cognitive development at baseline and during follow-up

**Fig. 1:**
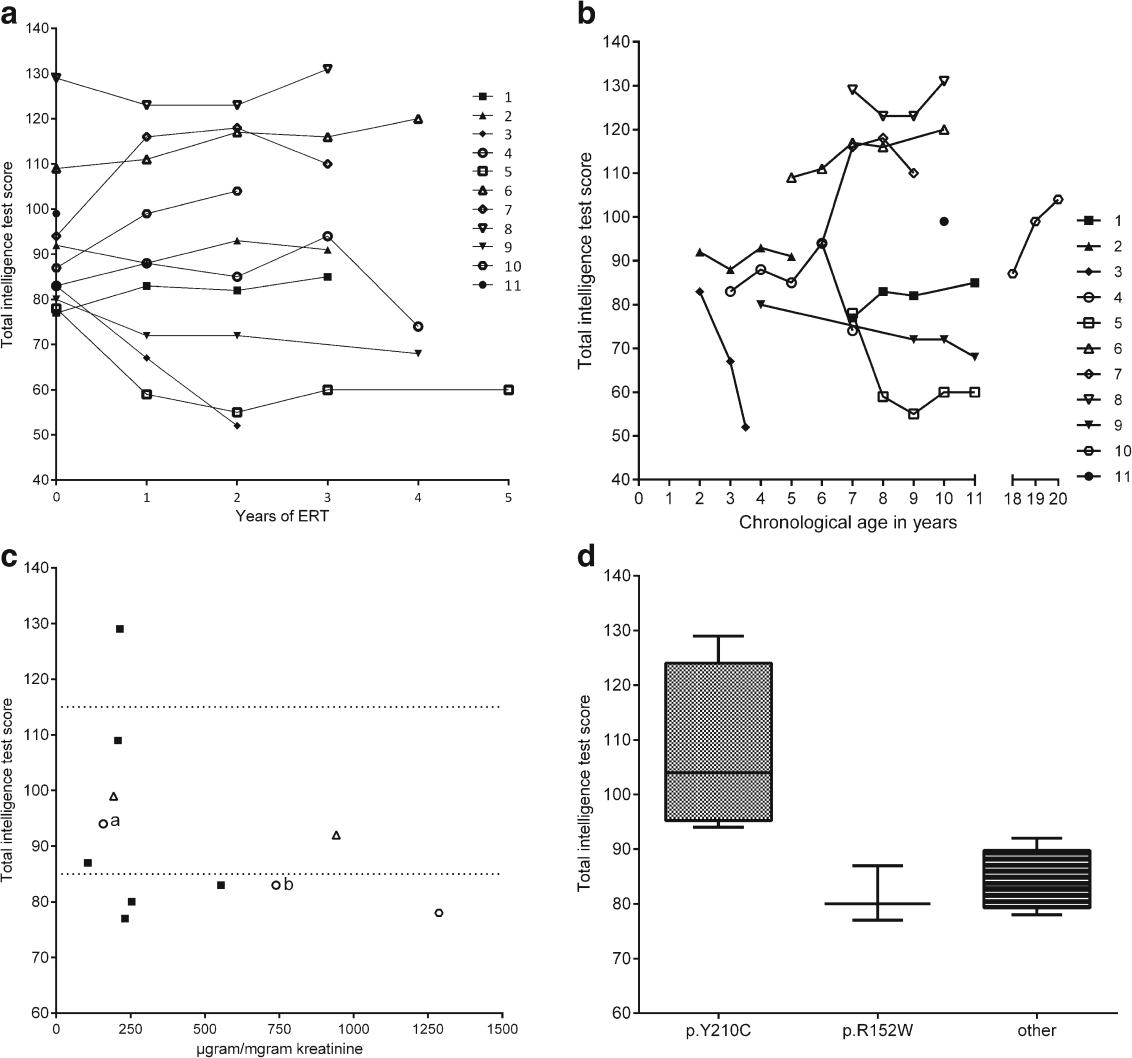
Cognitive development in 11 patients with Maroteaux-Lamy syndrome. (**a**) Long-term cognitive development expressed against duration of ERT, and (**b**) expressed against chronological age. The first assessment of patient 5 was based on one subscale score (the performance scale); due to shyness and withdrawn behavior, the other subscales were unreliable. (**c**) Patients’ total intelligence test scores at baseline versus urinary GAG level at baseline, relative to the siblings’ IQ scores. ■ = scores similar to siblings’, o = scores below siblings’, ∆ = no siblings. a = this child’s sibling had an extraordinarily high test score (IQ = 139). b = this child was patient 3 in Fig. 1a and b. (**d**) Total intelligence test scores related to mutation analysis—grouped by identified mutations

Forty-two intelligence tests were performed in the 11 patients (age range 2 to 20 years). Figure 1a shows the patients’ total IQ (TIQ) scores expressed against duration of enzyme therapy; Fig. 1b shows the total IQ scores expressed against chronological age. Six patients scored above the critical threshold of normal intelligence (>84) at all time points. At baseline, the test scores reflected levels of development that ranged from mildly delayed to above average; the group’s median intelligence test score was normal (87, range 73–129, n = 11). During follow-up, cognitive development remained stable (one year therapy median = 88, range 59–123, n =10; two years = 89, range 52–123, n = 10; three years = 94, range 60–131, n = 7). Expressed against chronological age, the scores also remained stable for the various age categories. However, there was one exception: over a period of 17 months from baseline, patient 3 (aged 1.9 at baseline) deteriorated over 30 points (2 standard deviations). His behavior was diagnosed as Autistic disorder. Until age 20 months, his skull size increased significantly (from 0 SD toward +2,5 SD) and he had frontal bossing and a dolichocephalic skull shape. While MRI showed prominent ventricles, lumbar puncture did not show raised intracranial pressure.

Disharmonic profiles were found in all patients and in 25 of the 42 tests performed; there was no consistency in disharmonic profiles.

### Education

Six of the 11 patients were attending or had successfully completed regular schools, three of whom required extra support (remedial teaching), due either to dyslexia (n = 2) or to mildly delayed IQ scores. The five other patients attended special schools for sick children (n = 3) or for children with motor disabilities (n = 2).

### Social and familial background

#### Siblings

Seven healthy siblings of seven patients agreed to be tested by a neuropsychologist as part of this study. The siblings of two other patients agreed only to inquiry of their school data. The remaining two patients had no siblings. The intervals between the patients’ tests and those of their sibling/siblings ranged between 5 and 9 months.

The siblings’ overall intelligence test scores were largely similar to the patients’ total test scores (Mann Whitney *U* Test: patients median 104 range 68–131; verbal 96 range 74– 25, performal 104 range 66–128, n = 7; siblings median 88 range 77–139; verbal 94 range 75–135, performal 84, range 81–150, n = 7). Five healthy siblings had harmonic profiles. The sixth sibling had a higher score in the verbal domain than in the performal domain; in the seventh it was the other way around.

Unfortunately, the siblings of the two patients with the lowest IQs (patients 3 and 5) were the ones who did not want to be tested. However, inquiry after their school results showed that these healthy siblings attended regular schools without needing special assistance; to date, they had never failed to be promoted to the next grade. On the basis of these school results, it was concluded that their intelligence was likely to be within the normal range (TIQ ≥ 85), unlike that of their affected siblings, whose scores indicated severe delays.

#### Parents

All parents’ highest parental education levels were obtained. These ranged from finishing primary school to obtaining a university degree. We found an association between the parents’ educational level and the level of the patients’ and their siblings’ test scores, most children of parents with a higher educational level having higher intelligence test scores and vice versa.

Seven of the 11 children had non-native speaking parents and were being raised bilingually. On average, patients of native Dutch-speaking parents had higher test scores (4/4 patients had normal to above normal intelligence at all times; range 94–131) than children with non-native-speaking parents (2/7 patients had normal test scores at all times; range 52–104).

### Age of diagnosis, GAG levels, genetic mutations

The disease was considered to be more severe in four patients (patients 2–5). They had presented at a young age (range of age at diagnosis 1.8–3.4 years) and excreted the highest of GAG levels in their urine at baseline (>300 μg/mg creatinine). Severe cardiomyopathy was present in three of the four patients. Figure 1c plots the patients’ GAG levels against their total IQ. Three of the four patients with high GAG levels (>300 μg/mg creatinine) had a mild or severe developmental delay (IQ < 85), against two of the seven patients with low GAG excretion.

Figure 1d categorizes the patients according to their mutations. The first group consisted of patients who were homozygous for the p.R152W mutation (n = 3, all of Turkish Ancestry). The second group had a p.Y210C mutation combined with either p.P313A or p.R327X (n = 4, all of Dutch Ancestry). The third comprised the patients with other mutations. Patients with the p.Y210C mutation had the highest total intelligence test scores.

### Brain abnormalities

During follow-up, all children had at least two brain MRI scans. Five had their first MRI before the start of ERT. Median age at the first MRI was 7.4 years (range 1.8–18.0) and median age at the second was 10.8 years (range 4.1–20.5). Median time between the two MRIs was 2.6 years (range 1.2–5.7). See Table 2 for a detailed description of the results.

**Table 2:** Results of baseline MRI and most recent MRI

		Rapid progressive		Slow progressive	
Domain		MRI 1	MRI 2	MRI 1	MRI 2
Enlarged Virchow Robin	Basal nuclei	2/4	2/4	3/7	4/7
	White matter	3/4	4/4	0/7	1/7
	Corpus Callosum	1/4	2/4	0/7	0/7
	Large lesions	1/4	1/4	0/7	0/7
White matter	Patchy lesion	4/4	2/4 ^1^	1/7	1/7
	Diffuse lesion	0/4	0/4	1/7	1/7
Ventricular enlargement	FOHWR	0/3	1/4	1/7	1/7
Intracranial pressure	Widened sinus rectus	3/4	3/4	2/7	3/7 ^2^
Atrophy	All fissures and sulci involved	0/4	0/3	0/7	0/7
Thinner corpus callosum		3/4	3/4	2/7	2/7
Mild compression of the spinal cord		3/4	3/4	2/7	3/7
Total abnormalities		20/43	21/43	12/77	16/77

^1^ White-matter abnormalities disappeared in two patients. In the remaining two patients, white-matter abnormalities decreased, but were still visible

^2^1/7 showed a slight increase, possibly due to differences in technique

Common findings at baseline were enlarged Virchow Robin spaces (in the basal ganglia and in the white matter); patchy white matter abnormalities (especially in the occipital area, but also in the frontal and periventricular area); widened sinus rectus, a thinner corpus callosum, and mild compression of the spinal cord.

**Fig. 2:**
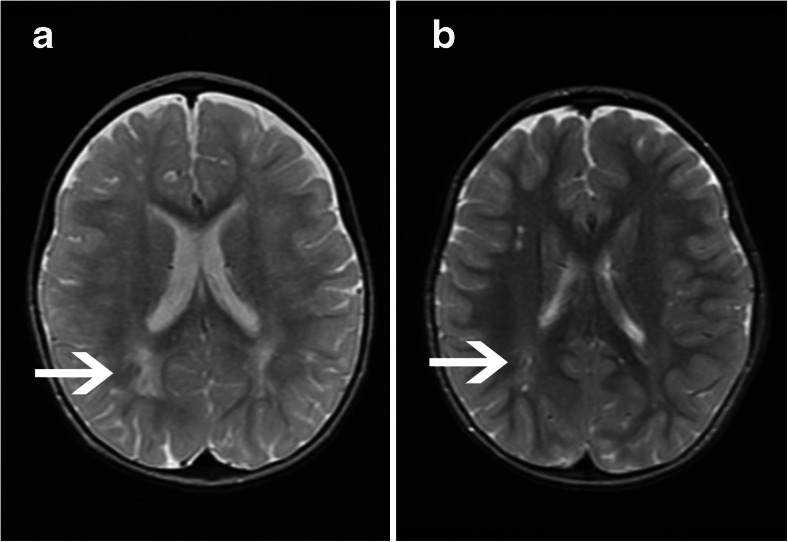
Longitudinal brain magnetic resonance imaging (MRI). (**a**) The patient was 2 years of age at first MRI. (**b**) The patient was 4.5 years old at the second MRI. The arrows indicate a delay in myelination in the occipital region (slices are at slightly different levels, due to the quality of MRI). Please note the presence of enlarged Virchow Robin spaces in the white matter in the second MRI (not present in the first MRI)

It is noteworthy that the patchy and diffuse white matter abnormalities became less intense over time and sometimes even disappeared (Fig. 2).

Brain abnormalities were found in a higher number of severely affected patients than in patients who were relatively mildly affected: 4/4 of the severely affected patients had Virchow Robin spaces in white matter vs. 1/7 of the mildly affected patients (MRI 2); 4/4 had patchy white-matter signal changes vs. 1/7 (MRI 1); and 3/4 had thin corpus callosum vs. 2/7 (both MRIs).

#### Miscellaneous

All patients visited an ophthalmologist and underwent hearing tests once and twice a year. Seven out of 11 patients needed glasses, all for hyperopia (4/4 were severely affected patients and 3/7 mildly affected patients). Four patients developed mild to moderate conductive hearing loss, and hearing adds were fitted and adjusted if necessary (range 7–43 dB right ear; 2–53 dB left ear) (Brands et al. 2013b; three of these patients were severely affected). At time of IQ testing, hearing and vision problems were sufficiently compensated (Table 1). Patients were regularly examined for carpal tunnel syndrome and five patients underwent carpal tunnel release. At the age of 5, patient 4 received a ventriculoperitoneal shunt for increased intracranial pressure. All patients required hospital admissions for medical procedures requiring general anaesthesia. The number of interventions ranged from 1 to 11, with a median of 5. The two most severely affected patients underwent 11 and 7 procedures.

## Discussion

To date, the extent to which MPS VI affects mental outcome has remained unclear. Our study shows that cognitive development in these patients is determined not only by familial and social-background factors, but, in patients with a severe form of the disease, also by the disease itself.

Based on the accumulated data of the literature and our study (Azevedo et al. 2013; Spranger et al. 1970; Vestermark et al. 1987), we conclude that, just as in other types of Mucopolysaccharidoses (Neufeld and Muenzer 2001), cognitive development in MPS VI presents as a spectrum from normal to delayed. However, the spectrum in MPS VI seems milder than in other mucopolysaccharidoses that affect the brain, such as MPS I, II, and III.

Whereas previous studies were cross sectional, our prospective approach found that cognition remained fairly stable in 90 % of our patients during follow-up periods that lasted up to 4.8 years. Progressive mental retardation was noted in only one of our 11 patients.

A number of variables influenced cognitive development in our patients with MPS VI. The most important factors appeared to concern social and family background. These patients were drawn from various cultural backgrounds and reflected a wide variation in parents’ educational level. Patients with non-native speaking parents generally had lower intelligence test scores when compared to those with native speaking parents. Also, as five of the 11 patients scored below average, three of whom had siblings with similar test scores and parents with relatively low educational levels, we conclude that social and familial background factors largely contributed to their relatively low cognitive level. In healthy children, cognition is known to be associated with parental educational level (Bradley and Corwyn 2002), inheritance (Bouchard and McGue 1981), and native and or non-native-speaking environments (Kort et al. 2005). Our study shows that any interpretation of cognitive test results should take into account social and familial background factors, especially when the patient population is small and diverse as in ultra-rare diseases such as MPS VI.

With regard to these social and familial background factors, we found that two patients clearly had lower test scores than expected. Both presented before the age of 5 years with severe symptoms and high GAG excretion in the urine (>300 μg/mg creatinine). While we concluded that MPS VI-related factors probably played a role in determining these children’s cognitive level, the picture was not clear-cut: at least one child with high GAG levels and early disease onset performed reasonably well and continued to do so during follow-up. We conclude that it is only in children with severe disease presentation, characterized by high urinary GAG excretion, that there seems to be a risk of low cognitive levels.

With regard to the effect of the genotype on cognition, we note that the group of patients with the p.Y210C mutation performed best (Karageorgos et al. 2007). These patients had parents who were all native-speakers of the national language, and who all had a high educational level. Similarly, they had siblings whose intellectual performance was in the highest range. Earlier, it was reported by Swiedler et al. (2005) that the p.Y210C mutation was associated with low GAG excretion and better growth. In three of the four children, p.Y210C was combined with p.R327X, a nonsense mutation leading to no production of the arylsulfatase B protein. This indicates that the second mutation did not influence the milder phenotype. Patients 3 and 5, who had the lowest intelligence test scores, both had severe mutations. One of these patients was homozygous for 1142 + 2 T > C, a mutation affecting the invariant splice donor sequence that is therefore expected to completely disrupt splicing; the other was homozygous for p.G324V, which leads to reduced synthesis of arylsulfatase B protein (Brands et al. 2013a).

Despite the seemingly limited influence of MPS VI on cognition at group level, there was a considerable need for special education services (73 % versus 5 % in the normal population). In part, this was no doubt explained by physical limitations such as limited joint mobility, bone deformities, pain, cardiac problems, and fatigue—medical problems that also substantially increased school absence. Also, the frequent hospitalizations and surgical procedures may have influenced cognitive ability. It is unsure whether educational potential was influenced by subtle neuropsychological deficits that had not been detected by standard intelligence tests. Although hearing problems and poor eye sight more often occur in severely affected patients, vision or hearing deficits were not a main contributor. At the time of testing, they appeared to have been sufficiently compensated by glasses and hearing aids.

The structural abnormalities detected on our patients’ brain MRIs were generally mild, fairly aspecific, and similar to those previously reported (Azevedo et al. 2013; Borlot et al. 2014; Palmucci et al. 2013; Rasalkar et al. 2011; Thorne et al. 2001; Vedolin et al. 2007; Zafeiriou and Batzios 2013). Patients with rapidly progressive and attenuated disease presentations had similar types of abnormality. Those with severe disease had slightly more abnormalities, particularly with regard to the frequency of white matter lesions (patchy lesions, thinner corpus callosum, and Virchow Robin spaces in the white matter). The white matter abnormalities found in our patient group were different from those in other lysosomal storage diseases such as Metachromatic Leukodystrophy (Gieselmann and Krageloh-Mann 2010) and Hunter Disease (Manara et al. 2011), in which such abnormalities are clearly progressive.

## Conclusion

Cognitive development in MPS VI presents in a spectrum that ranges from normal to delayed. Our study shows that cognitive development in MPS VI patients is determined not only by familial and social-background factors, but, in patients with a severe form of the disease, also by the disease itself. Cognition of patients with these hallmarks should be monitored closely. Furthermore, cognition testing in rare diseases such as MPS VI should include examination of social and familial background factors such as parental educational level, and testing of siblings’ cognitive levels. Such an approach is especially necessary in small patient populations from varied cultural backgrounds.

## Electronic supplementary material

ESM 1 (DOCX 15 kb)
