# Supplementary material for: Long-term cognitive follow-up in children treated for Maroteaux-Lamy syndrome
Source: J Inherit Metab Dis. 2015 Oct 8;39:285–92. doi: 10.1007/s10545-015-9895-8 (PMC4754322; doi:10.1007/s10545-015-9895-8)
Supplement: Supplementary file 1 — (DOCX 15 kb) [file 10545_2015_9895_MOESM1_ESM.docx]

**Supplement**

MRIs were scored for the presence or absence of the following parameters:

1.) Virchow robin spaces (VR ≤ 8 mm diameter) in the basal nuclei, white matter, and/or corpus callosum (CC) or large VR (≥ 8 mm diameter) in any location. Scores: 0 = absent; 1= present.

2.) Patchy and/or diffuse lesions in the white matter. Scores: 0 = absent; 1 = present.

3.) Ventricular enlargement. Scores: 0 = normal; 1 = deviant if the FOHWR index (frontal and occipital horn width ratio) was two or more standard deviations above the mean (Jamous et al 2003). Reference data were obtained from a control group of 20 healthy children analyzed at Erasmus MC (unpublished results).

4.) Increased flow void. Scores: 0 = non-dilated sinus rectus; 1= widened sinus rectus.

5.) Brain atrophy as measured by dilated subarachnoid spaces (SS). For this purpose, we measured the width of the Sylvian fissure and the interhemispheric fissure (measured at foramen of Monro level). Scores: 0 = brain atrophy absent if the width of the subarachnoid spaces was < 3 mm and none of the sulci were widened; 1= mild brain atrophy if the width of the spaces was ≥ 3 mm in the Sylvian fissure or interhemispheric fissure, but without widening of other fissures and sulci; 2 = significant cortical atrophy if the Sylvian fissure and interhemispheric fissures and other fissures and sulci were ≥ 3 mm and/or if there was significant loss of cortex and white matter.

6.) Compression of the spinal cord at the level of cranio-cervical junction. Scores 0 = absent or insignificant compression of the spinal cord; 1 = mild compression of the spinal cord (taping).

7.) Corpus callosum. Scores: 0 = normal; 1 = thinner than normal corpus callosum.
